# A Case of Desmoplastic Small Round Cell Tumor: Highlighting Diagnostic Challenges and Underscoring the Need for Standardized Treatment Protocols

**DOI:** 10.7759/cureus.70712

**Published:** 2024-10-02

**Authors:** Sydney Carpenter, Taylor Calicchia, Marcus Yoakam, Brett Dunbar

**Affiliations:** 1 Surgery, Kansas City University, Joplin, USA; 2 General Surgery, Cleveland Clinic Akron General, Akron, USA; 3 General Surgery, Ascension Via Christi Hospital, Pittsburg, USA

**Keywords:** desmoplastic small round cell tumors, ewsr gene, pelvic sarcoma, rare neoplasm, surgical case reports

## Abstract

Desmoplastic small round cell tumor is a rare and aggressive neoplasm, primarily affecting serosal surfaces within the abdomen and pelvic peritoneum in young males. Because of a low reported incidence, underdiagnosis and lack of current treatment guidelines pose a unique challenge for physicians and patients dealing with this disease. Therefore, it is imperative to present new information regarding this disease so that better detection and treatment guidelines can be created. This case report presents a 30-year-old male presenting with vague abdominal symptoms, ultimately diagnosed with desmoplastic small round cell tumor after image-guided biopsy. The ambiguity of initial symptoms highlights the diagnostic challenges associated with desmoplastic small round cell tumor, further complicated by the absence of standardized treatment guidelines. This report aims to raise awareness of this exceptionally rare cancer, contribute to the existing knowledge base, and emphasize the critical need for collaborative efforts to develop standardized treatment regimens.

## Introduction

Desmoplastic small round cell tumor is an uncommon neoplasm that represents a small subset of sarcomas with an incidence of around 450 cases documented since 1989 [1]. Arising from mesenchymal tissues, such as bone and connective tissue [2], they typically involve serosal surfaces of the abdomen and/or pelvic peritoneum. These tumors are exceptionally rare and mainly manifest in males during childhood or early adulthood. The prognosis is unfavorable due to their unique cell differentiation pattern, which leads to an aggressive tendency for metastasis [3]. Management for this condition involves extensive multidisciplinary treatments including chemotherapy (with or without stem cell rescue), surgical debulking of tumor, and radiation [4]. While desmoplastic small round cell tumor is a distinct entity within the sarcoma group, recent research has shown a shared gene fusion with Ewing sarcoma, the EWSR1-WT1 [5]. This discovery opens new avenues for treatment, potentially allowing insights from research of sarcomas with this genetic makeup to be applied to desmoplastic small round cell tumor, ultimately improving patient outcomes.

## Case presentation

A 30-year-old male presented to the emergency room with abdominal distension and pain described as intermittent cramping for three weeks. He reported experiencing decreased bowel movements and had tried laxatives without relief. The patient was able to produce infrequent bowel movements with pellet-like stool. He denied fever, nausea, and vomiting. There was no history of inflammatory bowel disease or family history of cancer. His past medical history was insignificant. On examination, the abdomen was distended but nontender. Basic labs were unremarkable, and urinalysis was negative for infection.

**Figure 1:**
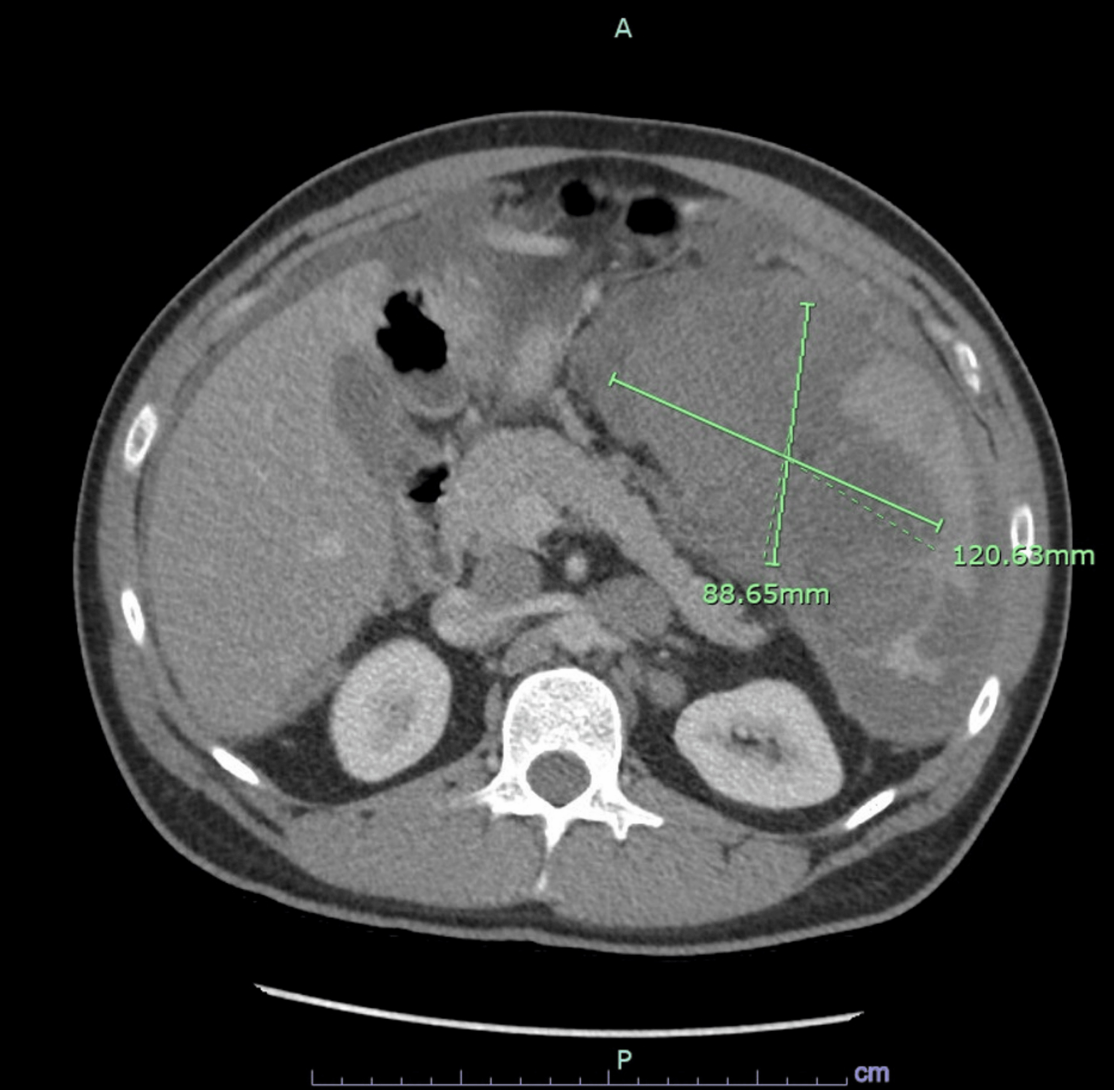
Initial computed tomography of abdomen and pelvis with contrast showing extensive focal and confluent lesions throughout the spleen with largest confluent mass measuring 12.1 cm.

**Figure 2:**
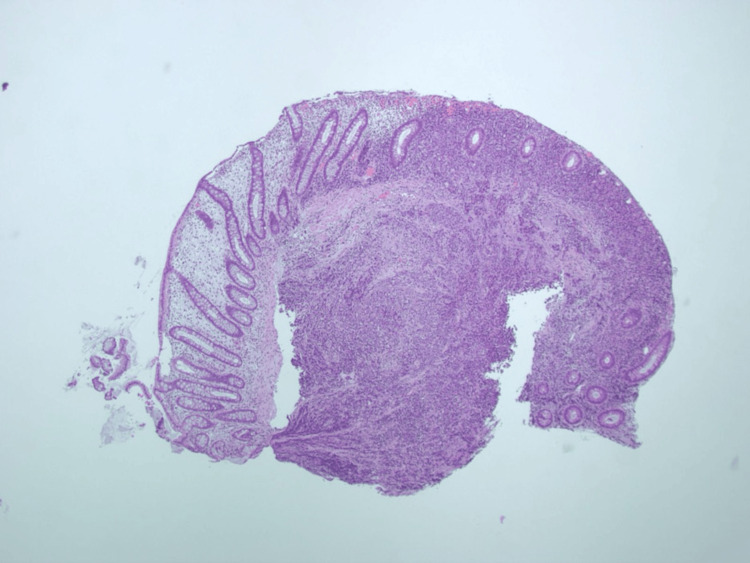
Colon biopsy at 20x with lamina propria and submucosal infiltrates with minimal desmoplastic stroma. Growth pattern is predominantly solid with some nested pattern.

A computed tomography scan of the abdomen and pelvis with contrast revealed splenic lesions, liver lesions, scattered lymphadenopathy, diffuse thickening of the mesentery, and moderate ascites concerning for peritoneal carcinomatosis (Figure 1). A flexible sigmoidoscopy was performed and revealed a rectal polyp for which a cold biopsy polypectomy was performed and the specimen was sent to pathology. Gross pathology of the specimen showed a solid pattern of growth with minimal to no desmoplastic stroma which did not suggest a diagnosis outright (Figure 2). Immunohistochemistry showed that the specimen was strongly positive for pancytokeratin and weakly positive for SATB2. The positive staining for SATB2 was confounding as that is typically associated with colonic adenocarcinomas.

**Figure 3:**
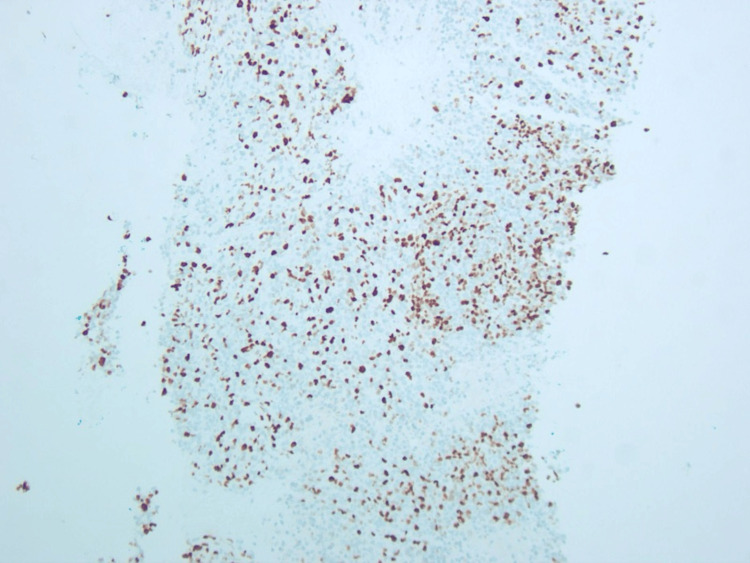
Ki-67 showing a high proliferation rate with staining in 40%-50% of viable tumor cells.

The patient then underwent a computed tomography-guided biopsy of abdominal mass with interventional radiologist in the following week. Immunohistochemical analysis of this mass was strongly and diffusely positive for CD56, a marker of neuroendocrine differentiation. The mass also was strongly positive for CD99 and focally positive for chromogranin and synaptophysin. The Ki-67 mitotic index was increased with 40% of cells staining positively (Figure 3). The submitting diagnosis at the time was high-grade neuroendocrine neoplasm.

**Figure 4:**
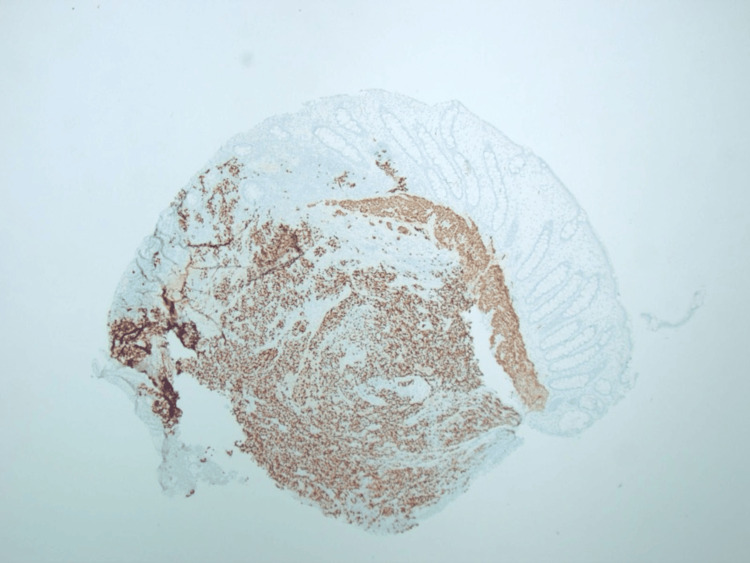
Strong and diffuse expression of desmin in tumor cells (20x).

The patient was referred to local medical oncology services, and Next Generation Sequencing tests on blood and tissue were ordered to identify potential targetable mutations. His local oncologist recommended a chemotherapy regimen consisting of carboplatin and etoposide [6], while Next Generation Sequencing was pending due to the bulky, diffuse metastasis and markedly high proliferative index. The patient had an internal jugular port implanted for systemic therapy and was started on treatment. The Next Generation Sequencing report returned and showed no actionable mutations, no mismatch repair deficiencies, and a programmed death ligand 1 (PD-L1) status of <1%. There was, however, a noted EWSR1-WT1 chromosomal arrangement, which is characterized by a translocation t(11;22)(p13;q12) that results in the EWSR1-WT1 fusion gene found in Ewing Sarcoma and desmoplastic small round cell tumor. This finding was brought to the attention of the pathologist who ran additional tests and was able to definitively diagnose desmoplastic small round cell tumor using this information and a strongly positive desmin stain (Figure 4).

Given the rarity and complexity of the disease, the patient’s local oncologist referred the patient to sarcoma specialists who recommended an initial positron emission tomography scan to evaluate the degree of the disease, a 17-week alternating chemotherapy regimen, used most commonly for Ewing Sarcoma, and positron emission tomography scans in between each cycle of chemotherapy to evaluate treatment response. The patient’s initial positron emission tomography scan demonstrated diffuse hypermetabolism involving the peritoneum with numerous enlarged lymph nodes in the porta hepatis and central retroperitoneum. There was also noted hypermetabolic lymphadenopathy in the right internal mammary chain, as well as a hypermetabolic lesion in the dome of the liver and a small left pleural effusion.

The patient was started on neoadjuvant chemotherapy treatment with a standard Ewing sarcoma regimen. The regimen consisted of alternating cycles of Vincristine, Doxorubicin, and Cyclophosphamide and Ifosfamide and Etoposide [7]. Doxorubicin was to be replaced by Actinomycin D after the ninth total cycle of chemotherapy in order to minimize potential cardiac toxicity. This is in line with the use of P6-protocols as described in the literature [8].

A positron emission tomography scan one month later showed improvement with decreased or diminished hypermetabolic adenopathy compared to prior scan. There was still significant hypermetabolic activity associated with the omentum and a moderate to large amount of ascites. The pleural effusion resolved at this time. Repeat positron emission tomography scan two months following aforementioned imaging did not show any significant or new changes when compared with previous imaging.

The patient has tolerated the chemotherapy well and has had no major side effects other than expected myelosuppression. This has been remedied with self-administered subcutaneous granulocyte-colony stimulating factor and occasional week-long breaks before cycles to allow blood cell count recovery. The patient’s treatment plan remains dynamic due to ongoing therapy, and treatment options will be reassessed following completion of the current regimen. Debulking surgery, radiation, and hyperthermic intraperitoneal chemotherapy were discussed as potential treatments in the multimodal approach to treating this rare cancer but will be contingent on the patient’s response to chemotherapy.

## Discussion

Desmoplastic small round cell tumor primarily presents with tumors of the serosal surfaces in the abdomen and pelvis in young males, and early diagnosis is difficult given the common presentation of non-specific gastrointestinal symptoms. At this time, desmoplastic small round cell tumor has been classified as an ultra-rare sarcoma with an incidence of ≤1 per 1,000,000 [9]. This extreme scarcity poses a significant challenge for diagnosis, treatment, and research. Our patient was seen with undefined, insidious presenting symptoms which likely reflected the large tumor burden ultimately reflecting late-stage disease. Early detection becomes particularly difficult when symptoms are non-specific, and the limited number of cases hampers the development of effective therapies but would be crucial due to the aggressive nature of the disease and limited effective treatment modalities for late stages.

Differentiating desmoplastic small round cell tumor from other small round cell tumors, such as Ewing sarcoma, gastrointestinal stromal tumor, neuroblastoma, and small-cell carcinoma, is crucial for guiding optimal treatment. Our patient's pathology findings posed a challenge to interpret. The specimen presented in the colonic mucosa and both the tumor and the soft tissue showed a solid pattern of growth with minimal to no desmoplastic stroma to suggest a definitive diagnosis. Staining also showed weak SATB2 expression, a tumor marker specific to colonic adenocarcinomas, further complicating the picture. Immunohistochemistry showed strong positivity for CD56 and CD99, along with focal positivity for chromogranin and synaptophysin. These findings are consistent with desmoplastic small round cell tumor presentation, although they are more typically found with neuroendocrine tumors [10].

Despite the aggressive nature of desmoplastic small round cell tumor, recent advancements in multimodality treatment strategies have shown promising results. However, the long-term prognosis remains guarded, with five-year overall survival rates still hovering around 10%-20% [11]. The current standard of care for desmoplastic small round cell tumor involves a multimodal approach involving neoadjuvant chemotherapy to shrink the tumor and improve surgical resectability, followed by cytoreductive surgery with hyperthermic intraperitoneal chemotherapy to maximize tumor removal and minimize the risk of local recurrence [12]. Postoperative chemoradiotherapy may further enhance local control and potentially improve survival, but current research cannot fully elucidate potential benefits. In line with current guidelines, our patient is undergoing 17 cycles of alternating chemotherapy with repeat positron emission tomography scans between every two cycles. The patient is currently tolerating therapy well and will continue to be monitored with the intent of eventually performing cytoreductive surgery with hyperthermic intraperitoneal chemotherapy sometime in the future followed by adjuvant radiation.

## Conclusions

This case report presents a 30-year-old male who initially presented with non-specific abdominal symptoms, ultimately diagnosed with desmoplastic small round cell tumor following extensive workup. Initial positron emission tomography scans indicate disease burden stabilization on intravenous chemotherapy regimen commonly used for Ewing sarcoma. The aggressive nature of desmoplastic small round cell tumor will necessitate an ongoing and dynamic multidisciplinary approach to treatment. Further research efforts should prioritize the development of evidence-based treatment protocols for desmoplastic small round cell tumor, incorporating optimal chemotherapy regimens, surgical approaches, and radiation therapy tailored to specific disease stages and patient characteristics. These advancements will be crucial for improving outcomes and achieving long-term disease control in this challenging malignancy.
